# Utility and validity of dynamic chest radiography in cystic fibrosis (dynamic CF): an observational, non-controlled, non-randomised, single-centre, prospective study

**DOI:** 10.1136/bmjresp-2020-000569

**Published:** 2020-03-25

**Authors:** Thomas Simon FitzMaurice, Paul Stephen McNamara, Dilip Nazareth, Caroline McCann, Ram Bedi, Matthew Shaw, Martin Walshaw

**Affiliations:** 1 Adult CF Unit, Liverpool Heart and Chest Hospital NHS Trust, Liverpool, UK; 2 Institute of Translational Medicine, University of Liverpool, Liverpool, UK; 3 Institute in the Park, Alder Hey Children’s Hospital, Liverpool, UK; 4 Institute of Infection and Global Health, University of Liverpool, Liverpool, UK; 5 Department of Radiology, Liverpool Heart and Chest Hospital NHS Trust, Liverpool, UK; 6 Department of Bioengineering, University of Washington, Seattle, Washington, USA; 7 Research Department, Liverpool Heart and Chest Hospital NHS Trust, Liverpool, UK

**Keywords:** cystic fibrosis, lung physiology, respiratory muscles, respiratory infection

## Abstract

**Introduction:**

Dynamic chest radiography (DCR) uses novel, low-dose radiographic technology to capture images of the thoracic cavity while in motion. Pulmonary function testing is important in cystic fibrosis (CF). The tolerability, rapid acquisition and lower radiation and cost compared with CT imaging may make DCR a useful adjunct to current standards of care.

**Methods and analysis:**

This is an observational, non-controlled, non-randomised, single-centre, prospective study. This study is conducted at the Liverpool Heart and Chest Hospital (LHCH) adult CF unit. Participants are adults with CF. This study reviews DCR taken during routine CF Annual Review (n=150), validates DCR-derived lung volumes against whole body plethysmography (n=20) and examines DCR at the start and end of pulmonary exacerbations of CF (n=20). The primary objectives of this study are to examine if DCR provides lung function information that correlates with PFT, and lung volumes that correlate whole body plethysmography.

**Ethics and dissemination:**

This study has received the following approvals: HRA REC (11 December 2019) and LHCH R&I (11 October 2019). Results are made available to people with CF, the funders and other researchers. Processed, anonymised data are available from the research team on request.

**Trial registration number:**

ISRCTN 64994816.

**Strengths and limitations of this study**

- Large sample size to assess current standard of care in cystic fibrosis (CF) management.
- Novel technology under assessment.
- To date, there have been no studies examining dynamic chest radiography in CF.
- Conducted in a specialist respiratory centre with a dedicated research department.
- Limited by the lack of non-CF controls.
- Limited by the non-randomised and non-controlled study design.

## Introduction and rationale

Dynamic chest radiography (DCR) is a new technology that captures radiographic imaging of the thoracic cavity while in motion, providing data on diaphragm excursion, chest wall movement and lung volumes, with a radiation dose much lower than fluoroscopic or CT scanning. It may also be better tolerated than traditional techniques for gathering pulmonary function data such as body plethysmography and spirometry, as it requires fewer forced manoeuvres and is quick to perform.

The DCR imaging machine installed at the Liverpool Heart and Chest Hospital (LHCH) has CE marking (73ED V.01, CE0197) and FDA 510(k) clearance (K182688), and it is in routine clinical use as part of the CF Annual Review (AR) process. DCR may be of use in assessing lung health in CF, a condition characterised by repeated exacerbations of lung disease associated with microbial infection and factors such as CF-related diabetes (CFRD), with a gradual decline of overall lung function.

## Background

### Dynamic chest radiography

DCR involves the use of a high-sensitivity flat-panel detector with a large field of view (the observable area of X-rays) to provide sequential radiographs at a much lower dose compared with conventional fluoroscopy and CT imaging.^1^ These sequential radiographs provide a moving image of the thorax over 10–20 s, at up to 15 images per second, without exposure to contrast agents. DCR has an effective dose of 0.125 mSv, compared with approximately 6.6 mSv for an unenhanced CT thorax.^1^ Although low-dose CT scanning is increasingly common and of sufficient quality to identify parenchymal disease, these images are static and may miss dynamic changes to moving structures seen on DCR.

Quantitative tools of DCR include a computer algorithm that automatically determines the borders of the diaphragm and lung apices and can track them over time. DCR can also be used to calculate lung volumes. This is done by acquiring both posteroanterior (PA) and lateral projections. Two-dimensional (2D) lung areas during maximal inspiration are automatically calculated and used to derive lung volumes.

The technology is particularly useful in observing moving structures with a high temporal resolution, such as the rapidly moving diaphragm or pulmonary vessels. DCR can detect diaphragmatic abnormalities such as discoordinated diaphragm motion, eventration or hemidiaphragm immobility. It can also visualise patterns of breathing to enhance understanding of underlying causes of dyspnoea^2–5^ and assessment of the severity of COPD.^6^

### Cystic fibrosis

CF is an autosomal recessive, multisystem disorder that arises from a defect in the CF transmembrane receptor (CFTR) protein. It affects around 10 500 individuals in the UK. The median lifespan with CF is now over 47 years, with cardiorespiratory disease the leading cause of mortality.^7^

In the lungs, dysregulation of ion transport by the defective CFTR protein leads to the build-up of thick secretions and a subsequent pro-inflammatory response.^8^ These thick secretions provide a favourable environment for chronic microbial colonisation by pathogens such as *Pseudomonas aeruginosa* and *Aspergillus fumigatus,* which represent risk factors for lung pathology and hospital admission.^9 10^ Repeated exacerbations of lung disease and eventual decline in overall lung function are common in CF. There is a well documented relationship between worsening lung function in CF and a change in breathing pattern and lung compliance.^11 12^ One factor that affects lung compliance is CFRD secondary to CFTR-mediated pancreatic dysfunction.

CFRD has a well-described negative impact on the lungs,^13 14^ possibly by inducing pulmonary oxidative stress leading to ‘stiffer’ less compliant lungs, and by its association with low body weight and poor nutritional status.^15^ Respiratory musculature such as the diaphragm may also function differently in CF compared to healthy individuals.^16 17^

### Assessment of pulmonary function

Assessment of pulmonary function is essential in the ongoing management of pulmonary diseases such as CF. These assessments usually consist of spirometry, gas transfer and lung volumes assessed by whole body plethysmography. The most common measurement of lung function in CF is forced expiratory volume of air in 1 s (FEV_1_). When compared with the results of other types of functional tests, spirometry may not detect early lung disease in CF.^18^

Lung compliance can be calculated by oesophageal pressure transduction, but this method is invasive and requires specialist skills and equipment. Its primary use is in the intensive care setting.^19^ Other experimental methods, such as photoplethysmography,^20^ exist although they have not reached routine use.

Diaphragmatic motion can be directly assessed by ultrasound, traditional fluoroscopy and MRI fluoroscopy. While ultrasound shows promise in areas such as weaning from mechanical ventilation^21^ and predicting recovery from non-invasive ventilation in COPD,^22^ or assessing the presence of diaphragm dysfunction during exacerbations of COPD,^23^ it requires skilled technicians and is not in widespread clinical use for assessing non-ventilated individuals. MRI fluoroscopy has shown correlation between diaphragmatic movement with FEV_1_ in people with COPD,^24^ but it is both time-consuming and resource-consuming to perform.

DCR is non-invasive and quick to perform and it provides information on moving structures in real time; it is a novel and potentially useful tool in improving our understanding of lung compliance, muscle function and lung disease in CF, which may be reflected by changes in diaphragm motion and breathing pattern seen on DCR. It may also provide an additional tool to whole body plethysmography for measuring lung volumes. The role of DCR in clinical practice may be in assessing changes to the function of respiratory mechanics as a result of CFRD, poor nutritional status and chronic microbial infection. Its rapid acquisition, low radiation dose and similar diagnostic abilities to standard PA chest X-ray (CXR) may be of clinical relevance as an adjunct tool to assess lung function during pulmonary exacerbations of CF.

## Study aims

### Primary objectives

The primary objectives of this study are to examine whether DCR provides quantitative motion analysis that correlates with pulmonary function testing, and lung volumes that correlate with volumes provided by body plethysmography.

#### Null hypothesis

Diaphragmatic movement as measured by DCR is not related to other measures of lung function, nor do lung volumes calculated by DCR correlate with those provided by whole body plethysmography.

#### Alternative hypothesis

DCR provides information on diaphragmatic movement that correlates with other measures of lung function as measured by spirometry or pulmonary function studies. The lung volumes calculated by DCR correlate with those provided by whole body plethysmography.

### Secondary objectives

Is diaphragmatic motion (excursion, velocity, recoil) measured by DCR related to microbial colonisation, the presence of CFRD, height, weight and body mass index (BMI)? Is DCR of clinical utility in CF?

## Methods and analysis

### Study setting

This study is conducted at LHCH, a large adult CF centre in North West England. The population is adults with CF who attend this centre. The frequent nature of contact with healthcare professionals in CF is anticipated to allow for several opportunities for recruitment.

### Part A: feasibility

This study partly involves the review of data collected during CF AR. Information gathered includes DCR (PA projection), spirometry and demographic information. DCR is routinely performed as part of the mandated CF AR at our CF centre. No information is lost, as DCR contains equivalent diagnostic information to a PA chest X-ray. Of a cohort of approximately 350 people with CF, we expect about 150 to meet inclusion criteria.

Spirometry is performed as a standard part of the CF AR. The following measures are recorded: FEV_1_ (l, % predicted), forced vital capacity (FVC) (l, % predicted) and FEV_1_/FVC ratio (% predicted). Spirometry is performed using Geratherm Spirostik spirometers (Geratherm, Bad Kissingen, Germany) and documented using Geratherm Blue Cherry software.

Diaphragm excursion and speed of motion over time are recorded from DCR. Key quantitative measures are:

- Diaphragmatic excursion (whole, maximal inspiratory, maximal expiratory) (mm).
- Peak diaphragm velocity at inspiration and expiration (mm.s^-2^).
- Peak distance from apex to diaphragm (mm).
- Lung-field area range during tidal breathing and lung-field area range during deep breathing (cm^2^).

These quantitative measures are also used in parts B and C of this study.

Derived values recorded by DCR are then compared against spirometry results and with regards to specific individual factors such as CFRD, microbial colonisation and physical characteristics (height, weight, BMI). We postulate that decreased diaphragmatic excursion and speed of motion correlate with reduced lung function as measured by FEV_1_, FVC and FEV_1_/FVC ratio and are associated with the presence of CFRD and pathognomonic CF microbes such as *P. aeruginosa*. Spirometry and/or pulmonary function testing forms the standard frame of reference in any ambiguous DCR reports, as it is for parts B and C of this study.

### Part B: validation

DCR in PA and lateral projections is used to calculate lung volume in n=20 individuals, against full lung function studies with volumes measured via whole body plethysmography:

- Estimation of lung volumes by DCR and combined lateral chest X-ray are compared against results provided by body plethysmography.
- Diaphragmatic excursion and velocity calculated by DCR are compared against pulmonary function testing with respect to pathophysiological factors known to affect lung function.

Whole body plethysmography is performed in accordance with ERS-ATS guidelines^25^ to give an estimate of lung volumes. This is then compared against total lung capacity (TLC) (l), as calculated by DCR. DCR TLC is calculated by selecting matched frames during maximal inspiration on both PA and lateral projections. Lung field area (cm^2^) in the 2D PA and lateral images is calculated, which is then combined to calculate TLC. The following measures are recorded from pulmonary function studies: FEV_1_(l), forced vital capacity (FVC) (l), FEV_1_/FVC ratio, PEF (l.min^-1^), functional residual capacity (FRC) (l), vital capacity (VC) (l), total lung capacity (TLC) (l), residual volume (RV) (l), TLC/RV (%), transfer factor (TL_CO_) (mmol.min^-1^.kPa^-1^), transfer coefficient (K_CO_) (mmol.min^-1^.kPa^-1^.l^-1^), alveolar ventilation (VA) (l), airways resistance (R_aw_) (kPa.s.l^-1^), specific airways resistance (sR_aw_) (kPa.s). Derived values recorded by DCR are then compared against full pulmonary function studies results and with regards to specific pathophysiological factors such as CFRD, microbial colonisation and physical characteristics (height (m), weight (kg), BMI (kg.m^-2^).

The correlation between lung-field area calculated from the PA DCR projection, and lung volumes calculated by both whole body plethysmography and DCR are compared in these 20 individuals.

### Part C: exacerbation

DCR imaging of a subgroup during CF pulmonary exacerbations (n=20).

- DCR and spirometry before (captured on CF AR), during (within 48 hours of admission) and at the end of an exacerbation (14 days, or on resolution of clinical signs and symptoms of infection).
- Correlation with clinical data such as antimicrobials, mucolytic therapy, duration of exacerbation, presence of CFRD and known microbial colonisation.

Antibiotics, microbial cultures, inhaled therapies and serum inflammatory markers are recorded.

For parts A, B and C of this study, all images are reported by an independent, qualified thoracic radiologist and reported via the local hospital imaging system. Any adverse findings noted during research review of the images are actioned by a member of the investigating team.

## Inclusion and exclusion criteria

### Inclusion criteria

- Age: ≥17 years old.
- Attending the adult CF Unit at LHCH.
- Confirmed CF diagnosis (positive sweat test, and by genotyping).
- Able to provide informed consent.

### Exclusion criteria

- Potentially pregnant or lactating.
- Refusal or inability to provide informed consent.
- Unable or unwilling to sit or stand to perform DCR in the radiology department.
- Unable or unwilling to follow tidal breathing instructions (eg, holding breath or taking a deep breath).
- Unable to perform reproducible spirometry and/or full pulmonary function studies within ATS-ERS criteria.
- Any serious or active medical or psychiatric illness, which, in the opinion of the investigators, would interfere with subject treatment, assessment or compliance with the protocol.
- For those in the validation part of this study: suffering an acute exacerbation of underlying CF.
- For those in the validation or exacerbation parts of this study: significant radiation exposure within the last year (eg, numerous CT scans of chest).
- Involved, either currently or recently; in other studies involved non-routine exposure to sources of ionising radiation.

## Statistical analysis plan

As this study is a preliminary, exploratory work with the aim of validation, no statistical power calculation has been carried out. The number of dynamic radiographs taken for this study is limited primarily by the size of our centre’s CF population. There are approximately 350 individuals with CF under the care of the Adult CF unit at LHCH. Of these, approximately 150 meet inclusion criteria and are expected to attend for AR while clinically stable during the study recruitment period. We intend to recruit 20 individuals each to parts B and C of this study as a realistic and pragmatic target for recruitment from the existing population at our CF centre. Moreover, this number would make the study feasible considering funding, effort and time involvement.

Statistical analysis is carried using the R software package, V.3.6.1, produced and supported by the R Foundation for Statistical Computing, under the GNU GPL v2 public license. All data are collated electronically and stored on a secure server in line with data protection (see section below). Data already collected from withdrawn individuals are included, unless otherwise stated. Two-sided p values of <0.05 are considered as statistically significant. Of note, 95% CI is presented where appropriate and feasible.

Comparison of the data is primarily between DCR results and pulmonary function data in individuals. Depending on the distribution of the data set, paired t-test or the relevant non-parametric equivalent is used to analyse within groups. Categorical data are presented as frequency and percentage. Continuous data are presented as number of observations, mean, SD, median and IQR as appropriate.

## Methodological issues

The observational nature of this study and the high degree of contact that individuals with CF have with healthcare professionals allows a relatively large number of individuals to be recruited with relative ease from the clinical environment. A potential limitation to this study is the lack of non-CF controls. However, exposing a cohort of otherwise healthy individuals to ionising radiation precluded their use. DCR-derived measures of diaphragm excursion and velocity in healthy individuals have already been published and compared with individuals with Chronic Obstructive Pulmonary Disease (COPD).^6 26^ Furthermore, it is questionable how useful such a control group would be given that muscle action in people with CF (even those with good lung function) may differ significantly from that in healthy individuals.^16 17^

## Patient and public involvement

Individuals with CF and their families/friends have been consulted in the outpatient clinic and inpatient environment by members of the investigating team during the design of this study. The response to receiving DCR in lieu of standard chest X-ray during AR has been positive. Patient feedback on when and how to attend the department has been taken into account when designing the study in order to reduce the burden of additional testing and pursue the least restrictive options for conducting research in this cohort.

## Data management and oversight

### Anonymity

Any patient-identifiable information is removed from any published or presented data. For recording of patient information, participants are assigned a study-specific unique identifying number which is used instead of their name or hospital record number in data analysis and reporting.

### Data management

Data gathered include DCR image sequences, pulmonary function/plethysmography results, clinical data and biochemical results, with the latter available from the hospital electronic patient records system. All images are stored and backed up via the local PACS system, and the unprocessed X-ray data are stored on a secure LHCH server. These data are stored securely within hospital facilities in accordance with the Data Protection Act 2018. No personally identifiable information is stored outside of hospital servers.

### Blinding

At the point of image analysis, the images are anonymised to provide single blinding from the team member conducting the analysis of the images. This is done within the DCR imaging software.

Any information that comes to light and directly impacts a particular individual (eg, deterioration in lung function found on spirometry, or abnormal findings on review of DCR images) is immediately acted on by a clinical member of the investigating team, and the individual informed as soon as realistically appropriate.
